# Risk factors for deep surgical site infection following surgically treated peri-ankle fractures: a case–control study based on propensity score matching

**DOI:** 10.1186/s13018-022-03436-3

**Published:** 2022-12-15

**Authors:** Haitao Zhao, Jinghong Meng, Tao Sun, Zihan Wan, Shiji Qin, Fengqi Zhang, Zhiyong Hou

**Affiliations:** 1 Department of Orthopaedic Trauma Center, The 3rd Hospital of Hebei Medical University, No 139 Ziqiang Road, Shijiazhuang, 050051 Hebei People’s Republic of China; 2 Department of Foot and Ankle Surgery, The 3rd Hospital of Hebei Medical University, Shijiazhuang, 050051 Hebei People’s Republic of China; 3 Department of Rheumatology and Immunology, The 3rd Hospital of Hebei Medical University, Shijiazhuang, 050051 Hebei People’s Republic of China; 4 Department of Bone Tumor, The 3rd Hospital of Hebei Medical University, Shijiazhuang, 050051 Hebei People’s Republic of China; 5 College of Basic Medicine, Hebei Medical University, Shijiazhuang, 050000 Hebei People’s Republic of China

**Keywords:** Peri-ankle fractures, Surgical site infection, Epidemiology, Risk factor, Propensity score matching

## Abstract

**Aims:**

This study aims to identify the risk factors for deep surgical site infection (DSSI) following surgically treated peri-ankle fractures.

**Methods:**

We performed a retrospective case–control study using the propensity score matching (PSM) method in 1:2 ratio, based on the 6 baseline variables, including age, gender, living area, insurance type, fracture location and surgical date. Data on patients who underwent surgical treatment of peri-ankle fractures were collected by inquiring their hospitalization medical records and operative records, as well as the laboratory reports. Conditional logistic regression analysis was performed to identify the risk factors for DSSI.

**Results:**

A total of 2147 patients were eligibly included and 74 had a DSSI, indicating an incidence rate of 3.4%. After PSM, 70 cases of DSSI and 140 controls without DSSI were matched, constituting the study cohort. The univariate analyses showed significant differences between groups in terms of history of any surgery, time to operation, surgical wound classification, smoking, alcohol drinking, RBC count, hemoglobin concentration and hematocrit (%). The conditional logistic regression analysis showed time to operation of < 4 or > 9 (vs 4–9 days); unclean wound, current smoking, high-energy injury mechanism and lower hematocrit were independent risk factors for DSSI.

**Conclusions:**

Timely modification of smoking and hematocrit (%), and limiting operation within a rational time frame for an optimized soft tissue condition, may provide potential clinical benefits for SSI prevention.

## Introduction

Surgical site infection (SSI) remains a major issue after foot and ankle trauma, especially peri-ankle joint fractures (ankle, calcaneus, and talus), although antibiotics are routinely administered before surgery. As was reported, the overall rate of SSI was 3.0–25.0%, with deep SSI (DSSI) varying from 1.3 to 6.8% [1–6]. These DSSIs generally required multiple surgical procedures for allowing facilitation of bone and soft tissue union, but sometimes, the results are not encouraging. Ovaska et al. [3] reported a 27% treatment failure rate of DSSI, defined as that necessitated surgical debridement in patients with ankle fractures. Furthermore, even if timely and appropriately managed, these DSSIs still led to serious consequences such as need for multiple, permanent joint dysfunction, amputation and even death [7], also extended the total hospital stay by above 200% and increased the healthcare costs by 300% [8, 9].

Identification of risk factors of DSSIs, especially the modifiable, and targeting them with preventive or therapeutic interventions remain the most cost-effective method. In the previous studies, numerous risk factors for DSSI after surgically treated peri-ankle fractures were identified, including open fracture [4, 10], obesity [5, 10–14], comorbid diabetes [4, 15], peripheral neuropathy [4], higher American Society of Anesthesiologists (ASA) grade [6, 12, 14], smoking tobacco [2, 13, 14, 16], alcohol overuse [5], wound classification [10, 12, 14], prolonged surgical duration [2, 11, 17], delayed operation [1, 11, 13, 18], surgeon volume or experience [10, 11, 19], hypoalbuminemia [1, 11, 20], severer fracture type [16], high-energy injury mechanism [10], bone allografting [1], drain usage [6, 21], perioperative pyrexia [21] and postoperative non-compliance [4]. However, the limited sample size and relatively limited variables included for adjustment might have compromised the findings. Particularly, it is of crucial importance in balancing the baseline characteristics between cases and controls for adequate adjustment for confounders.

In this study, we designed it as a matched case–control study, with 1:2 ratio to match the cases with controls using propensity score matching (PSM), currently a most popular method. Our hypothesis is that there are still some un-identified factors that may affect the incidence of DSSI following surgically treated peri-ankle fractures.

## Methods

This was designed as a retrospective case–control study in accordance with the principles outlined in the Helsinki Declaration. The study protocol was approved by the Ethics Committee of The Third Hospital of Hebei Medical University, which waived the need for informed consent from participants, due to the de-identified data used.

### Identification of cases

From January 2015 to December 2021, adult patients (aged 18 years or older) who had undergone operative treatment for peri-ankle fractures (ankle, intra-articular calcaneus, or talus fractures) were included.

The inclusion criteria were adult patients with acute closed peri-ankle fracture surgically treated within 21 days of injury, who had complete data of interest and at least 12-month follow-up records. The exclusion criteria were open fracture, old fracture, multiple trauma, concurrent fractures of other locations, conservative treatment, preoperative existence of infections, postoperative superficial site infection, loss or inadequate (< 12 months) follow-up assessments, or incomplete data of interest.

Patients who developed a deep surgical site infection (DSSI) within 12 months postoperatively were included in case group. The diagnosis of surgical site infection (SSI) and classification was in accordance with the criteria proposed by the Center for Disease Control and Prevention [22]. A DSSI refers an infection involving the deep soft tissues and must meet at least one of the following: persistent wound discharge or dehiscence, abscess or gangrenosis requiring surgical interventions (debridement, implant exchange or removal).

### Propensity score matching

By inquiring the electronic medical charts, 74 cases and 2073 controls were identified. For minimizing the effects of confounders, these 74 cases were matched with controls (with 2073 as candidates) in a ratio of 1:2, using the propensity score matching (PSM) method, based on the following variables: age, sex, living place (rural or urban), operation date (converted to a specific number, e.g., May 12/2018, as 43232), insurance type (public or non-public), and fracture location (ankle, intra-articular calcaneus, talus).

The matching principle and procedure were as follows: calculation of the propensity score for each patient by using a multivariate logistic model based on the above 6 variables, use of the greedy algorithm (nearest neighbor without replacement) with a caliper width of 0.02 to optimize matching. Standardized mean difference (SMD) was used to assess the post-matched balance between groups, with a value ≥ 0.15 suggestive of imbalance.

The R software 3.6.5 (R Foundation for Statistical Computing, Vienna, Austria) was used for PSM.

### Data collection and variables of interest

All data were collected by inquiring the hospitalization medical charts, operative records and laboratory testing reports. The variables of interest included body mass index (BMI) calculated by dividing weight in kilogram by square of height in meter, current smoking or alcohol drinking, comorbidities (hypertension, diabetes, cerebrovascular disease, heart disease, liver disease, renal disease), history of any surgery, injury-related data (injury mechanism, fracture type based on AO classification system according to the fracture location, articular surface involvement and fracture severity; time from injury to operation, accompanied dislocation), surgery-related data (surgical wound classification, anesthesia pattern, ASA classification, reduction pattern, hardware implanted, bone grafting, surgical duration, intraoperative bleeding, allogeneic transfusion) and laboratory results (plasma albumin, fasting blood glucose, hemoglobin, hematocrit, red blood cell (RBC) count, platelet count, plateletcrit, white blood cell (WBC) neutrophils, and lymphocyte count) preoperatively. Specifically, for patients who had multiple measurements preoperatively, the one closest to the index operation was applied to eliminate the potential time-dependent effects. Surgical wound cleanliness was classified according to the classification system proposed by the Center for Disease Control and Prevention [22]: I, clean; II, clean/contaminated; III, contaminated; and IV, dirty.

### Statistical analyses

For after-PSM case and control group, we, first, used univariate analyses to evaluate the between-group differences. Continuous variable was expressed with mean and standard deviation (SD) or median and interquartile range (IQR), based on whether the data were normally distributed (by Shapiro–Wilk test). Student’s *t* test or Mann–Whitney *U* test was used, as appropriate. Categorical variable was expressed with frequency and percentage, and the difference was detected by chi-square test or Fisher’s exact test, as appropriate.

Then, variables that were tested with *P* value ≤ 0.10 in the univariate analyses were further entered into the multivariate conditional logistic regression analysis to identify the independent factors for DSSI. The relative ratio (RR) with 95% confidence interval (95% CI) was used to indicate the association magnitude. *P* < 0.05 was set as the statistical significance level. The R software 3.6.5 (R Foundation for Statistical Computing, Vienna, Austria) was used for all analyses.

## Results

In the present study, a total of 3375 patients were initially included, and 1228 were excluded on the basis of above predefined exclusion criteria: open fracture (212), old fracture (79), multiple trauma (61), concurrent fractures of other locations (218), conservative treatment (128), preoperative existence of infections or signs (23), postoperative superficial site infection (137), loss or inadequate (< 12 months) follow-up assessments (197), or incomplete data of interest (173). The remaining 2147 patients constituted the source dataset, including 74 (3.4%) DSSIs and 2073 (96.6%) non-DSSIs. There were 1575 (73.4%) male and 572 (26.6%) female patients, and the mean age was 42.0 years (SD, 12.8 years; range, 18–88 years). According to fracture location, 1111 (51.7%) had an ankle fracture, 930 (43.4%) had a calcaneal fracture and 106 (4.9%) had a talus fracture. About two-thirds (66.5%, 1428/2147) had a public medical insurance for expense reimbursement.

**Fig. 1:**
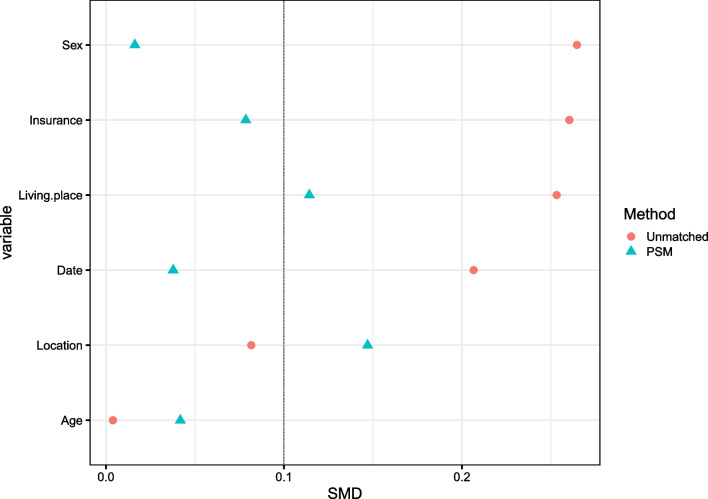
Comparison of SMD between before and after PSM

Table 1 shows the differences of baseline variables based on which propensity score was calculated and PSM was conducted before and after PSM. Compared to those who did not develop a DSSI, patients with a DSSI were more living in rural areas (73.0% vs 61.2%, *P* = 0.041) and were more males (83.8% vs 73.0%, *P* = 0.040). As for other variables (age, surgery date, insurance and fracture location), no significant difference was observed (all *P* > 0.05), despite with a SMD of 0.207 and 0.262 for surgical date and insurance pattern, respectively. After PSM, all the 6 variables were not significantly different (all *P* > 0.05), and the corresponding SMD was less than predefined level. Figure 1 depicts the change of SMD before and after PSM for the 6 baseline variables.

**Table 1:** Comparison of the baseline characteristics of the infection and non-infection patients before and after PSM

Variable	Before PSM				After PSM			
	Non-infection (*n* = 2073)	Infection (*n* = 74)	SMD	*P*	Non-infection (*n* = 140)	Infection (*n* = 70)	SMD	*P*
Age (median [IQR])	41 (32, 51)	39 (31.3, 53)	0.004	0.735	43 (32.5, 53)	39 (32, 54.5)	0.042	0.593
Date (median [IQR])	42970 (42604, 43291)	42903 (42352, 43278.5)	0.207	0.131	42927 (42582, 43185)	42947.5 (42451.8, 43299.3)	0.038	0.658
Public insurance (*n*, %)	1384 (66.8)	44 (59.5%)	0.262	0.189	87 (62.1)	43 (61.4)	0.079	0.920
Living place (rural, %)	1268 (61.2)	54 (73.0)	0.253	0.041	94 (67.1)	50 (71.4)	0.114	0.528
Sex (male, %)	1513 (73.0)	62 (83.8)	0.265	0.040	110 (78.6)	58 (82.9)	0.016	0.464
Fracture location (%)			0.082	0.759			0.147	0.793
Ankle	1073 (51.8)	38 (51.4)			69 (49.3)	35 (50.0)		
Calcaneus	899 (43.4)	31 (41.9)			64 (45.7)	30 (42.9)		
Talus	101 (4.9)	5 (6.8)			7 (5.0)	5 (7.1)		

IQR, interquartile range; PSM, propensity score matching; SMD, standardized mean difference

Table 2 shows the between-group difference in terms of history of any surgery (31.4% vs 15.7%), time to operation (in continuous variable, 7.3 ± 5.4 vs 5.9 ± 3.1; and in categorical variable, *P* < 0.001), surgical wound classification (clean-contaminated, 25.7% vs 5.7%), smoking (30.0% vs 16.4%), alcohol drinking (55.7% vs 40.7%), lower RBC count (in continuous variable, 4.1 ± 0.7 vs 4.3 ± 0.6; and in categorical variable, 35.7% vs 22.1%), hemoglobin concentration (in continuous variable, 124.5 ± 22.6 vs 133.6 ± 18.5; and in categorical variable, 32.9% vs 14.3%) and hematocrit (in continuous variable, 36.8 ± 6.5 vs 39.5 ± 5.3; and in categorical variable, 62.9% vs 43.6%); however, in terms of other variables, no significant difference was observed.

**Table 2:** Univariate analysis of variables between infection and non-infection group after PSM

Variable	Infection group (*n* = 70)	Non-infection group (*n* = 140)	*P*
Obesity	16 (22.9)	18 (12.9)	0.064
Dislocation	7 (10.0)	15 (10.7)	0.873
Hypertension	15 (10.7)	11 (15.7)	0.300
Diabetes	24 (17.1)	11 (15.7)	0.793
Cerebrovascular disease	9 (6.4)	5 (7.1)	0.845
Heart disease	5 (3.6)	3 (4.3)	0.799
Liver disease	2 (1.4)	2 (2.9)	0.475
Renal disease	0	1 (1.4)	0.156
History of any surgery	22 (31.4)	22 (15.7)	0.008
Interval from injury to operation (days)	7.3 ± 5.4	5.9 ± 3.1	0.016
4–10	33 (47.1)	105 (75.0)	< 0.001
< 4	12 (17.1)	10 (7.1)	
> 10	25 (35.7)	25 (17.9)	
Surgical wound classification			
Type I (clean)	62 (74.3)	132 (94.3)	< 0.001
Type II (clean–contaminated)	18 (25.7)	8 (5.7)	
Anesthesia pattern			0.110
Regional	52 (74.3)	117 (83.6)	
General	18 (25.7)	23 (16.4)	
Injury mechanism			0.076
Low to medium energy	13 (18.6)	42 (30.0)	
High energy	57 (81.4)	98 (70.0)	
Fracture type based on AO classification system			0.990
44A	21 (30.0)	39 (28.1)	
44B	10 (14.3)	25 (18.0)	
44C	7 (10.0)	12 (8.6)	
81A	1 (1.4)	1 (0.7)	
81B	2 (2.9)	4 (2.9)	
81C	2 (2.9)	5 (3.6)	
82B	10 (14.3)	16 (11.5)	
82C	17 (24.3)	37 (26.6)	
Current smoking	21 (30.0)	23 (16.4)	0.023
Alcohol drinking	39 (55.7)	57 (40.7)	0.040
Reduction method			0.868
Closed	7 (10.0)	13 (9.3)	
Open	63 (90.0)	127 (90.7)	
Internal fixation hardware			0.579
Plate/screw	55 (78.6)	104 (74.3)	
Screw only	11 (15.7)	30 (21.4)	
Others	4 (5.7)	6 (4.3)	
Bone grafting			0.474
Yes	7 (10.0)	10 (7.1)	
No	63 (90.0)	130 (92.9)	
Surgical duration (minutes)	143.4 ± 64.0	137.1 ± 62.1	0.495
≥ 180 min	21 (30.0)	29 (20.7)	0.136
Intraoperative blood loss (ml)	214.4 ± 272.0	178.4 ± 261.5	0.354
≥ 300 ml	17 (24.3)	23 (16.4)	0.172
ASA classification			0.315
I	11 (15.7)	27 (19.3)	
II	49 (70.0)	102 (72.9)	
III–IV	10 (14.3)	11 (7.9)	
Allogeneic transfusion	2 (1.4)	4 (5.7)	0.079
BMI (kg/m^2^)	26.1 ± 4.1	25.8 ± 3.8	0.639
Obesity (BMI ≥ 30)	16 (22.9)	18 (12.9)	0.064
Albumin (g/L)	39.6 ± 7.5	40.9 ± 4.8	0.141
< 35	13 (18.6)	14 (10.0)	0.080
FBG (mmol/L)	5.9 ± 2.1	5.8 ± 1.4	0.620
≥ 6.1	16 (22.9)	39 (27.9)	0.437
RBC (*10^12^/L)	4.1 ± 0.7	4.3 ± 0.6	0.047
< Lower limit	25 (35.7)	31 (22.1)	0.036
Hemoglobin (g/L)	124.5 ± 22.6	133.6 ± 18.5	0.002
< Lower limit	23 (32.9)	20 (14.3)	0.002
Hematocrit (%)	36.8 ± 6.5	39.5 ± 5.3	0.002
< Lower limit	44 (62.9)	61 (43.6)	0.008
Platelet count (*10^9^/L)	251.2 ± 117.3	238.3 ± 76.4	0.344
> 300	14 (20.0)	21 (15.0)	0.359
Plateletcrit (%)	0.21 ± 0.11	0.19 ± 0.06	0.168
< 0.16	18 (25.7)	29 (20.7)	0.413
WBC count (*10^9^/L)	**9.0** ± 2.6	8.9 ± 3.0	0.795
> 10	23 (32.9)	43 (30.7)	0.753
Neutrophil count (*10^9^/L)	6.4 ± 2.3	6.5 ± 2.9	0.975
> 6.3	23 (41.4)	64 (45.7)	0.556
Lymphocyte count (*10^9^/L)	**1.6** ± 0.6	1.6 ± 0.8	0.658
< 1.1	15 (21.4)	28 (20.0)	0.809

Bold indicates Infection group: patients with an SSI; Non-infection group: patients without an SSI

BMI, body mass index; WBC, white blood cell count; FBG, fasting blood glucose; ASA, American Society of Anesthesiologists RBC, red blood cells, reference range: male, 4.0–5.5 × 10^12^/L, female, 3.5–5.0 × 10^12^/L. Hemoglobin, male, ≥ 120 g/L, female, ≥ 110 g/L; hematocrit, reference range: male, 40–50%; female, 35–45%

Table 3 shows the conditional logistic regression analysis results that five variables were significantly associated with increased risk of DSSI, including time from injury to operation (< 4 and > 9 vs 4–9 days, RR = 5.2, 2.4, respectively), wound classification (IIvs I, RR = 4.4), current smoking (RR = 2.6), injury mechanism (high- vs low energy, RR = 2.1) and hematocrit (< lower limit vs normal range, RR = 1.9).

**Table 3:** Conditional logistic regression analysis of surgical delay and intraoperative variables

Variables	RR and 95%CI	*P*
Interval between injury and operation (days)	1.86 (1.19–2.91)	
4–10	Reference	
< 4	5.2 (1.8–14.9)	0.002
> 10	2.4 (1.1–5.1)	0.028
Wound classification (IIvs I)	4.4 (1.7–11.7)	0.003
Current smoking	2.6 (1.2–5.6)	0.014
Injury mechanism (high vs low and medium energy)	2.1 (1.1–4.8)	0.036
HCT (< lower limit)	1.9 (1.0–3.9)	0.047

## Discussion

In this study, we identified five risk factors that were independently associated with increased risk of DSSI, including time to operation of < 4 and > 9 relative to 4–9 days, unclean wound, current smoking, high-energy injury mechanism and lower hematocrit. These results suggest that special attention should be paid to certain patient groups, since patients suffering from high-energy injure and having an unclean wound are clearly at high risk of DSSI. It is especially reassuring that three factors were modifiable, i.e., the time to operation, smoking and lower hematocrit. This means that modifying these risk factors is possibly achievable and could potentially translate into improved outcome by reducing incidence of DSSI.

We reported a rate of DSSI of 3.4%, which was in range (1.3–6.8%) in the literature [1–6]. The varying rates reported were attributable to multiple aspects, relating to wide definition of DSSI, study patient characteristics, sample size, less or overrepresentation of this injury, and length of follow-up period. For example, in the study by Ovaska et al. [3], the authors included open fractures, defined deep infection as an infection necessitating at least one surgical debridement, and had a substantially longer follow-up time (up to 57 months), collectively leading to a 5.0% rate of DSSI. In contrast, we used the standard criteria for definition of DSSI proposed by the CDC guideline and excluded the open fracture, multiple trauma or concurrent fractures and others. However, a concern might arise that the overrepresentation of complex fracture and medically complicated patients (e.g., having multiple comorbidities and hemodynamic instability) referred from inferior hospitals might have affected the representativeness of DSSI rates.

Our result supported high-energy injury mechanism as a significant factor for DSSI, as it increased the risk by 2.1-fold. This finding was also confirmed by previous studies [10, 11, 23], which found a 2.6- to 7.5-fold increased risk of DSSI conferred by high-energy trauma. We believed the explanation for this relationship lied in the fracture severity and the surrounding soft tissue envelop damage directly resulting from high-energy trauma, which thus generally required more surgical trauma (namely, the second “hit”) and prolonged surgical time. Clearly, these would contribute to an increased risk of exposing bacterial colonization and wound infection. Similarly, the unclean wound site, largely attributable from the high-energy trauma (traffic trauma, fall from a height, or mechanical accident), would increase the risk of DSSI [10, 12, 14]. These both factors were determined upon the fracture occurred, unmodifiable, and should be at least kept in mind when evaluating the risk of DSSI.

The role of smoking in wound or bone union complications has been repeatedly confirmed, and the mechanisms have been well established [2, 13, 14, 16]. Smoking was prevalent in approximately 20–25% of general population and 30–50% of males [24], especially the manual workers, who represented a predominant population predisposed to peri-ankle fractures. Therefore, quitting smoking could provide certain benefits in wound infection complications. In a randomized controlled trial, Sorensen et al. [25] demonstrated that even 4 weeks of abstinence from smoking would reduce the risk of wound infection by 86.2%; and in another study of calcaneal fractures, cessation of smoking even for 5 days preoperatively would contribute to a positive effect on the rate of postoperative wound healing [26]. Therefore, we encourage every smoker to quit smoking upon the admission, and even a reduction in number of cigarettes or short-duration quitting smoking would provide a beneficial effect, especially compromised soft tissue cases [27].

The lower hematocrit was also found to be associated with increased risk of DSSI in this study, in line with the previous reports. Willis et al. [28] found the increased 2.38-fold risk of DSSI and 3.05-fold risk of organ space infection associated with preoperative lower hematocrit, but not for superficial infection, following 98,869 cases of total hip arthroplasty. Keswani et al. [29] demonstrated that lower preoperative hematocrit was not only an independent risk for 30-day complications including deep wound infection, but also increased the 30-day unplanned readmission or severe adverse event by 5.24 times following revision shoulder arthroplasty. Specific at trauma, lower hematocrit may represent an acute blood loss status after fracture, also may reflect the concurrent medical condition or nutritional status. Anyhow, it is prudent to appropriately delay surgery, or consider addition of an iron supplement or erythropoietin to restore blood stores prior to surgery [28, 30].

The timing of peri-ankle fracture surgery undoubtedly has a significant effect on wound complications. However, the cutoff value cited in previous studies is varying in < 8 h, 24 h, 48 h, 4 day, 5 days and 6 days, with positive, negative or neutral results [18, 31–33]. Schepers et al. [18] suggested the operation for closed ankle fracture to be preferably treated within the first day in their case series. In our study, we divided patients into three groups based on risk of DSSI for each day additionally delayed to operation and found the lowest rate when operation was conducted at 4–9 days of injury; in comparison, either earlier or later surgery was associated with higher risk of infection. Due to the fact that very few surgeries were performed within 24 s in our institution, we could not capture the potential benefits of within-24-h surgery. Our finding supported the conventional teaching recommendation of delaying 5–7 days until swelling has subsided completely to operate to facilitate soft tissue vitality and minimize related complications [34], but with a wider time frame (4–9 days) in ours, which may be related to the overrepresentation of the fracture severity in a level I trauma center.

The limitations to this study should be mentioned. First, the retrospective design, an inherent limitation, would have affected the precise of results. Since DSSI is uncommon, a prospective study design is not feasible. Second, the data might have been compromised in accuracy and precise, which was a general limitation of only reliance on medical and operative charts for data collection, because patient data were not always properly documented. Third, smoking was identified as an independent risk factor, but the quantification (frequency and number per day) was not captured, thus not allowing providing more specific quitting smoking comments. Fourth, like every multivariate analysis, the residual confounding effects from unmeasured, unconsidered, or unknown variables remain. Fifth, this study was conducted in a tertiary referral hospital with a level I trauma center; thus, the fracture severity or patient condition complexity was overrepresented, and the findings should be cautiously treated.

In conclusion, we found a 3.4% rate of DSSI following surgeries of closed peri-ankle fractures, and five factors, including three modifiable ones, were identified to independent factors for DSSI. Recognition of these factors would facilitate the surgical management of peri-ankle fractures; timely modification of smoking and lower hematocrit, and rationally controlling operation within a time frame for an optimized soft tissue condition, may provide potential direct benefits.

## Data Availability

All the data will be available upon motivated request to the corresponding author of the present paper.

## Abbreviations

DSSI: Deep surgical site infection
PSM: Propensity score matching
SMD: Standardized mean difference
BMI: Body mass index
ASA: American Society of Anesthesiologists
RBC: Red blood cell
WBC: White blood cell
FBG: Fasting blood glucose
IQR: Interquartile range
RR: Relative ratio
CI: Confidence interval
